# Cardiovascular risk and renal injury profile in subjects with type 2 diabetes and non-albuminuric diabetic kidney disease

**DOI:** 10.1186/s12933-023-02065-2

**Published:** 2023-12-13

**Authors:** Maurizio Di Marco, Sabrina Scilletta, Nicoletta Miano, Nicola Marrano, Annalisa Natalicchio, Francesco Giorgino, Stefania Di Mauro, Agnese Filippello, Alessandra Scamporrino, Paola Tribulato, Giosiana Bosco, Francesco Di Giacomo Barbagallo, Roberto Scicali, Agostino Milluzzo, Teresa Ballirò, Lucia Frittitta, Pietro Castellino, Francesco Purrello, Salvatore Piro, Antonino Di Pino

**Affiliations:** 1 Department of Clinical and Experimental Medicine, University of Catania, Catania, Italy; 2 Department of Precision and Regenerative Medicine and Ionian Area, Section of Internal Medicine, Endocrinology, Andrology and Metabolic Diseases, University of Bari Aldo Moro, 70124 Bari, Italy

**Keywords:** Type 2 diabetes, Diabetic kidney disease, Non-albuminuric diabetic kidney disease, Cardiovascular risk, Arterial Stiffness, Renal resistive index, Urinary biomarkers

## Abstract

**Background:**

In the last years, the classical pattern of diabetic kidney disease (DKD) has been partially overcome, because of the uncovering of a new DKD phenotype with significant renal dysfunction without presence of albuminuria: the non-albuminuric DKD (NA-DKD). To date, the cardiovascular risk associated with this phenotype is still debated. We investigated the cardiovascular risk and renal injury profile of NA-DKD subjects in comparison with other DKD phenotypes.

**Methods:**

Pulse wave velocity (PWV), intima-media thickness, presence of carotid atherosclerotic plaque, renal resistive index (RRI), and a panel of urinary biomarkers of kidney injury were evaluated in 160 subjects with type 2 diabetes, stratified according to estimated glomerular filtration rate (eGFR) and urinary albumin to creatinine ratio (UACR) into four groups: controls (UACR < 30 mg/g and eGFR ≥ 60 mL/min/1.73 m^2^), A-DKD (Albuminuric-DKD, UACR ≥ 30 mg/g and eGFR ≥ 60 mL/min/1.73 m^2^), NA-DKD (UACR < 30 mg/g and eGFR < 60 mL/min/1.73 m^2^), AL-DKD (Albuminuric and Low eGFR-DKD; UACR ≥ 30 mg/g and eGFR < 60 mL/min/1.73 m^2^).

**Results:**

Subjects with NA-DKD showed a higher PWV (11.83 ± 3.74 m/s vs. 10.24 ± 2.67 m/s, *P* = 0.045), RRI (0.76 ± 0.11 vs. 0.71 ± 0.09,* P* = 0.04), and prevalence of carotid atherosclerotic plaque (59% vs. 31%, *P* = 0.009) compared with controls. These characteristics were similar to those of subjects with AL-DKD, whereas the profile of A-DKD subjects was closer to controls. After multiple regression analyses, we found that RRI, that is in turn influenced by eGFR (β = − 0.01, *P* = 0.01), was one of the major determinants of PWV (β = 9.4, *P* = 0.02). Urinary TreFoil Factor 3, a marker of tubular damage, was higher in NA-DKD subjects vs. controls (1533.14 ± 878.31 ng/mL vs. 1253.84 ± 682.17 ng/mL, *P* = 0.047). Furthermore, after multiple regression analyses, we found that urinary osteopontin was independently associated with PWV (β = 2.6, *P* = 0.049) and RRI (β = 0.09, *P* = 0.006).

**Conclusions:**

Our data showed a worse cardiovascular and renal injury profile in NA-DKD subjects. This finding emphasizes the central role of eGFR in the definition of cardiovascular risk profile of diabetic subjects together with albuminuria.

## Background

Diabetic kidney disease (DKD) is a complication that occurs in about 40% of patients with type 2 diabetes and it is the leading cause of end-stage renal disease in developed countries [1]*.*

In the last few years, the classical pattern of DKD with the progressive worsening of albuminuria and the subsequent decline of estimated glomerular filtration rate (eGFR) has been partially overcome because of the evidence of different patterns of DKD [2]. Traditionally, the diagnosis of DKD was based on the detection of albuminuria (Albuminuric DKD, A-DKD), which is an essential marker of renal damage in diabetes. However, recent epidemiological studies have uncovered a subgroup of patients with diabetes who exhibit significant renal dysfunction without elevated levels of urinary albumin excretion—Non-albuminuric DKD (NA-DKD). Indeed, several studies have shown a decreased prevalence of A-DKD and an increased prevalence of NA-DKD as the current trend in DKD epidemiology [3–5].

Several factors may have contributed to the rising prevalence of NA-DKD: a higher prevalence of hypertension and obesity, a reduction in smoking and the use of multifactorial interventions leading to improved glucose, blood pressure, and lipid management [6].

NA-DKD is a challenging clinical entity as it is rather poorly understood and often misdiagnosed because of its atypical presentation. To date, it is not clear whether there are differences in the cardiovascular risk and renal injury profile of patients affected by NA-DKD. Data in the literature have reported conflicting results: studies on type 1 and type 2 diabetes have shown that both albuminuria and low eGFR are independently associated with cardiovascular and renal events; however, they provide conflicting estimates of outcome rates associated with different phenotypes. In a post-hoc analysis of the Action in Diabetes and Vascular disease: preterAx and diamicroN modified release Controlled Evaluation (ADVANCE) study the risk for cardiovascular events was lower in patients with normoalbuminuria and stage 3 chronic kidney disease (CKD) than in those with albuminuria and stage 2 CKD [7]; in contrast, a post-hoc analysis of the Fenofibrate Intervention and Event Lowering in Diabetes (FIELD) study showed that the non-albuminuric phenotype was associated with a higher risk of cardiovascular death, compared to microalbuminuria with an eGFR > 60 mL/min/1.73 m^2^ and macroalbuminuria with eGFR > 90 mL/min/1.73 m^2^ [8], and accordingly, in the Renal Insufficiency and Cardiovascular Events (RIACE) study all-cause mortality risk was higher in the non-albuminuric group with eGFR < 45 mL/min/1.73 m^2^ than in the group with albuminuria alone [9].

This finding raises serious concerns about the pathophysiology and mechanisms underlying the elevated cardiovascular risk found in NA-DKD patients. In the last few years, interest in identifying biomarkers that may improve diagnosis and risk stratification has increased. These biomarkers should capture inflammation, fibrosis, glomerular and tubular involvement, and mitochondrial impairment [10–12]. Additionally, biomarkers could be coupled with kidney structure evaluation obtained by abdominal ultrasound, which is a non-invasive procedure that can provide information about renal morphology and vascularity.

The aim of this study was to investigate the cardiovascular risk profile of subjects with NA-DKD compared with other DKD phenotypes; our fist aim was to investigate early markers of vascular damage such as arterial stiffness and carotid atherosclerosis. In addition, we characterized the renal profile of these patients with ultrasound measurements of intrarenal resistive index (RRI) and renal volume (RV); finally, we investigated the association between a panel of urinary markers strictly linked to renal injury.

## Methods

### Study subjects

One hundred sixty subjects with type 2 patients who attended our university hospital for diabetes and cardiovascular risk evaluation were recruited in this study. The inclusion criterion was an age range 45–75 years. The exclusion criteria were malignancies, rheumatological diseases, drug abuse, and corticosteroids therapy. All patients underwent a physical examination and review of clinical history, smoking status, and medications.

Body mass index (BMI) was calculated as weight (kg)/[height (m)]^2^. Blood pressure (BP) was measured with a calibrated sphygmomanometer after 10 min resting. Venous blood samples were drawn from the antecubital vein on the morning after an overnight fast. Baseline venous blood samples were obtained for the measurement of clinical biochemistry parameters. LDL cholesterol concentrations were estimated using the Friedewald formula. A sample of spot urine was collected.

### Biochemical analyses

Plasma glucose, serum total cholesterol, triglycerides, and HDL cholesterol were measured using available enzymatic methods [13].

HbA_1c_ was measured via high performance liquid chromatography using a National Glycohemoglobin Standardization Program and was standardized to the Diabetes Control and Complications Trial (DCCT) [14] assay reference. Chromatography was performed using a certified automated analyzer (HLC-723G7 hemoglobin HPLC analyzer; Tosoh Corp.) (normal range 4.25%–5.9%).

Albuminuria determination was performed as albumin-to-creatinine ratio (UACR) in a spot urine sample and the mean of two different values obtained in a period of 3–6 months was considered [15].

Estimated glomerular filtration rate (eGFR) was assessed with the Chronic Kidney Disease Epidemiology Collaboration (CKD-EPI) equation [16].

### Study groups

Subjects were assigned to four different groups according to UACR and eGFR as follows: controls (UACR < 30 mg/g and eGFR ≥ 60 mL/min/1.73 m^2^), A-DKD (UACR ≥ 30 mg/g and eGFR ≥ 60 mL/min/1.73 m^2^), NA-DKD (UACR < 30 mg/g and eGFR < 60 mL/min/1.73 m^2^), AL-DKD (Albuminuric and Low eGFR DKD; UACR ≥ 30 mg/g and eGFR < 60 mL/min/1.73 m^2^).

### Carotid ultrasound examination

Ultrasound scans were performed using a high-resolution B-mode ultrasound system. All ultrasound examinations were performed by a single physician who was blinded to the clinical and laboratory characteristics of the patients. Longitudinal B-mode (60 Hz, 128 radiofrequency lines) images of the right common carotid artery 2 cm below the carotid bulb were obtained using a high-precision echo tracking device (MyLab Alpha, Esaote, Maastricht, NL) paired with a high-resolution linear array transducer (13 MHz) to acquire quality intima-media thickness (qIMT) using the built-in echo tracking software.

### Pulse wave velocity

The SphygmoCor CvMS (AtCor Medical, Sydney, Australia) system was used for the determination of the pulse wave velocity (PWV) [17]. This system uses a tonometer and two different pressure waves obtained at the common carotid artery (proximal recording site) and at the femoral artery (distal recording site). An electrocardiogram was used to determine the start of the pulse wave. The PWV was determined as the difference in travel time of the pulse wave between the two different recording sites and the heart, divided by the travel distance of the pulse waveform. The PWV was calculated on the mean basis of 10 consecutive pressure waveforms to cover a complete respiratory cycle.

### Pulse wave analysis

All measurements were made from the right radial artery by applanation tonometry using a Millar tonometer (SPC-301; Millar Instruments, Houston, TX, USA). The measurements were performed by a single investigator with the subject in the supine position. The data were collected directly with a desktop computer and processed with SphygmoCorCvMS (AtCor Medical, Sydney, Australia). The aortic waveform has two systolic pressure peaks, the second is caused by wave reflection from the periphery. With arterial stiffening, both the PWV and the amplitude of the reflected wave are increased such that the reflected wave arrives earlier and adds to (or augments) the central systolic pressure. The aortic waveform in the pulse wave analysis was subjected to further analysis for the calculation of the aortic Aug and AugI (calculated by dividing augmentation by pulse pressure). Pulse pressure is the difference between the systolic and diastolic BPs [18].

### Renal Resistive Index (RRI)

RRI was derived from the Doppler spectrum of intrarenal arteries as the difference between maximum (peak systolic) and minimum (end-diastolic) flow velocity to maximum flow velocity, using a high-precision echo tracking device (MyLab Alpha, Esaote, Maastricht, NL) paired with a convex transducer [19]. The RRI was calculated as the mean of 3 different measurements.

### Renal volume (RV)

RV of the right kidney was calculated with the ellipsoid formula derived from the cranio-caudal, mediolateral, and anteroposterior diameters, using a high-precision echo tracking device (MyLab Alpha, Esaote, Maastricht, NL) paired with a convex transducer. Values were adjusted for body surface area (BSA) [20]*.*

### Quantification urinary renal function biomarkers

The BioPlex^®^ (Bio-Rad, Hercules, California, USA) platform was used for the determination of urinary biomarkers. The commercial package Bio-Plex Pro™ RBM Human Kidney Toxicity Assay Panel 2 (albumin, β2 microglobulin [u-B2M], cystatin C [u-Cys], Neutrophil Gelatinase-Associated Lipocalin [u-NGAL], osteopontin [u-OPN], and TreFoil Factor 3 [u-TFF3]) was used to evaluate the protein content in the urinary samples, according to the manufacturer’s protocol. Briefly, urine samples were thawed on ice and, after centrifugation at 500×*g* for 5 min, were diluted 1:50. After blockade of nonspecific binding sites, 30 µL of standards, controls or diluted samples were added to 96-well plates and incubated with 10 µL of fluorescently dyed magnetic microspheres covalently coupled to specific antibodies for the desired biomarkers. The plates were incubated for 1 h at RT and washed three times with wash buffer. Afterward, 40 µL of biotinylated detection antibody was added to the wells and the plates were incubated for an additional hour at RT. The final detection complex was completed with the addition of conjugated streptavidin–phycoerythrin. The median relative fluorescence units from the antibody reactions were acquired using a BioPlex 200 analyzer (Bio-Rad) and BioPlex Manager Software™ version 6.2 Build 175 (Bio-Rad).

### Statistical analyses

The sample size was calculated based on PWV using a level of significance (α) set to 5% and a power (1 − β) set to 80%. The estimated sample size was 38 patients per group.

Statistical comparisons of clinical and biomedical parameters were performed using Stat View 6.0 for Windows. The data are presented as mean ± SD or median (interquartile range). Each variable’s distributional characteristics, including normality, were assessed using the Kolmogorov–Smirnov test. One-way ANOVA for clinical and biological data was performed to test the differences among groups, and the Bonferroni post hoc test for multiple comparisons was also performed. The χ^2^ test was used for categorical variables. A *P* value < 0.05 was considered significant. When necessary, numerical variables were logarithmically transformed to reduce skewedness.

To identify variables independently associated with variations of PWV and RRI, we performed three multivariate regression models: the first model included cardiovascular risk factors (age, sex, BMI, smoking status, systolic BP [SBP], history of cardiovascular Disease [CVD]); variables reaching significance in the first model were included in a second model including biochemical variables (LDL and HDL cholesterol, HbA1c, eGFR, UACR > 30 mg/g); finally, variables reaching significance in the second model were included in a third model with renal variables (RRI—not in the analysis for RRI -, u-B2M, u-Cys, u-NGAL, u-OPN, and u-TFF3). The same models were used to perform a multiple logistic regression analysis to identify variables independently associate with the presence of atherosclerotic plaques.

Furthermore, we performed a multivariate logistic regression model in order to identify variables related to alterations in PWV and RRI. We used a cut-off of 10 m/s for PWV and 0.70 for RRI [21, 22]. The considered independent variables were age, sex, smoking status, BMI, being in secondary prevention, PAS, use of sodium-glucose cotransporter-2 inhibitors (SGLT-2i), use of Angiotensin Converting enzyme inhibitors (ACEi) or Angiotensin Receptor Blockers, HbA1c, eGFR, UACR > 30 mg/g.

The variance inflation factor (VIF) was used to check for the problem of multicollinearity among the predictor variables in multiple regression analysis. Any variable with a VIF that exceeded 4 was excluded from the model. No variable was detected with a VIF greater than 4.

The study was approved by the local ethics committee (Comitato Etico Catania 2, protocol n. 270/C.E. 26 April 2022). Informed consent was obtained from each participant.

## Results

### Study population characteristics

According to the eligibility criteria, 160 patients were included in this study. The study population was divided into the following four groups (based on UACR and eGFR levels): 40 subjects without DKD (Controls), 41 subjects with albuminuria (A-DKD), 41 subjects with eGFR reduction without albuminuria (NA-DKD), and 38 subjects with both albuminuria and eGFR reduction (AL-DKD). As shown in Table 1, the four groups were homogeneous for blood pressure, fasting glycemia and LDL cholesterol. Age was significantly higher in NA-DKD and AL-DKD patients. The groups were homogeneous also for the number of patients with history of CVD (myocardial infarction, stroke and peripheral artery disease), with the exception of the AL-DKD group, which showed a significantly higher percentage of subjects with a history of CVD in comparison with controls and the NA-DKD group. In the study population there was a major prevalence of males, especially in A-DKD patients. Regarding medications, we found statistically significant differences among the groups, with a greater use of metformin in controls and the A-DKD group and a higher use of SGLT-2i in AL-DKD group, as expected from clinical indications.

**Table 1:** Clinical and metabolic characteristics of the study population according to UACR and eGFR

	Controls (*n* = 40)	A-DKD (*n* = 41)	NA-DKD (*n* = 41)	AL-DKD (*n* = 38)
Age, years	63.45 ± 7.56	62.78 ± 7.15	68.19 ± 6.30*†	69.56 ± 4.91*†
Sex, no. (%) of females	27 (67)	37 (90)^*^	27 (66)^†^	30 (79)
Active smokers, no. (%)	11 (27)	19 (46)	10 (24)^†^	10 (26)
History of CVD^§^, no. (%)	7 (17)	12 (29)	7 (17)	17 (45)^*‡^
BMI, kg/m^2^	28.73 ± 5.26	30.48 ± 5.20	27.69 ± 3.09^†^	27.72 ± 3.79^†^
SBP, mmHg	132.62 ± 15.97	132.75 ± 11.38	132.75 ± 11.38	136.36 ± 8.13
DBP, mmHg	77.75 ± 9.80	81.87 ± 12.44	78.00 ± 8.15	77.23 ± 8.58
HbA_1c_, % (mmol/mol)	7.2 ± 1.3 (56 ± 14)	7.5 ± 1.5 (58 ± 16)	6.8 ± 0.9^†^ (51 ± 10)^†^	7.1 ± 1.2 (54 ± 14)
Fasting glucose, mg/dL	135.82 ± 39.53	136.76 ± 36.53	128.58 ± 49.92	125.94 ± 34.99
eGFR, mL/min/1,73 m^2^	91.38 ± 14.35	90.65 ± 11.57	50.78 ± 7.93^*†^	48.95 ± 14.05^*†^
UACR, mg/g	11 (8.5–16.5)	66 (45–279.5)^*^	11 (7–17)^†^	93 (47.5 – 284.5)^*†^
Total cholesterol, mg/dL	160.87 ± 39.76	149.66 ± 29.78	144.27 ± 30.81^*^	156.15 ± 36.55
HDL cholesterol, mg/dL	47.77 ± 12.51	45.83 ± 13.10	43.56 ± 10.91	48.23 ± 11.75
LDL cholesterol, mg/dL	88.44 ± 33.13	80.52 ± 28.37	74.78 ± 26.05	79.22 ± 37.34
Triglycerides, mg/dL	104 (82–162.5)	104 (86.75–147)	105 (81–156)	133 (111–175)
Medications				
Metformin, no. (%)	39 (97)	37 (90)	25 (61)^*†^	23 (61)^*†^
Insulin, no. (%)	13 (33)	14 (34)	14 (34)	19 (50)
SGLT2 inhibitors, no. (%)	8 (20)	15 (37)	17 (41)^*^	20 (53)^*^
DPP4 inhibitors, no. (%)	4 (10)	1 (2)	4 (10)	4 (11)
GLP1-RAs, no. (%)	18 (45)	19 (46)	24 (59)	16 (42)
Sulphonylureas, no. (%)	2 (5)	-	2 (6)	-
Antithrombotics, no. (%)	25 (63)	23 (56)	24 (59)	16 (42)
Statins, no. (%)	26 (65)	35 (85)^*^	32 (78)	29 (76)
ACE-i/ARBs, no. (%)	23 (57)	30 (73)	32 (78)^*^	29 (76)

Data are presented as mean ± SD, median (IQR), or percentage. A-DKD: albuminuric diabetic kidney disease (UACR ≥ 30 mg/g and eGFR ≥ 60 mL/min/1.63 m^2^); NA-DKD: non-albuminuric diabetic kidney disease (UACR < 30 mg/g and eGFR < 60 mL/min/1.63 m^2^); AL-DKD: albuminuric and low estimated glomerular filtration rate diabetic kidney disease (UACR ≥ 30 mg/g and eGFR < 60 mL/min/1.63 m^2^); CVD: cardiovascular disease; BMI: body mass index; SBP: systolic blood pressure; DBP: diastolic blood pressure; eGFR: estimated glomerular filtration rate; UACR: urinary albumin to creatine ratio; SGLT2: sodium-glucose transporter 2; DPP4: dipeptidyl-peptidase 4; GLP1-RAs: glucagon like peptide 1—receptor agonists. ACE-i: angiotensin converting enzyme inhibitors; ARBs: Angiotensin receptor blockers. ^§^history of CVD includes myocardial infarction, stroke, and peripheral artery disease. *P < 0.05 versus group A; ^†^*P* < 0.05 versus group B; ^‡^*P* < 0.05 versus group C

### Cardiovascular profile of the study population according to UACR and eGFR

As shown in Table 2, patients with NA-DKD showed higher PWV compared with controls (11.83 ± 3.74 m/s vs. 10.24 ± 2.67 m/s, *P* = 0.045); no differences were observed between patients with NA-DKD and AL-DKD (11.83 ± 3.74 m/s vs. 11.99 ± 3.98 m/s, *P* = 0.86). In the simple regression analysis PWV was associated with age (r = 0.28; *P* = 0.0012), eGFR (r = − 0.22; *P* = 0.01) and RRI (r = 0.36; *P* < 0.0001).

**Table 2:** Cardiovascular and renal profile of the study population according to UACR and eGFR

	Controls (*n* = 40)	A-DKD (*n* = 41)	NA-DKD (*n* = 41)	AL-DKD (*n* = 38)
PWV, m/s	10.24 ± 2.67	10.64 ± 3.62	11.83 ± 3.74^*^	11.99 ± 3.98^*^
AugI, %	29.30 ± 9.99	32.00 ± 12.01	28.36 ± 6.26	31.83 ± 9.48
Carotid plaque, no. (%)	12 (31)	17 (41)	24 (59)^*^	28 (74)^*†^
qIMT, mm	0.81 ± 0.14	0.81 ± 0.15	0.83 ± 0.12	0.86 ± 0.11
RRI	0.71 ± 0.09	0.71 ± 0.08	0.76 ± 0.11^*†^	0.77 ± 0.08^*†^
RV/BSA (mL/m^2^)	80.15 ± 24.08	91.45 ± 29.94	78.57 ± 17.04^†^	84.12 ± 26.67

Data are presented as mean ± SD or percentage. A-DKD: albuminuric diabetic kidney disease (UACR ≥ 30 mg/g and eGFR ≥ 60 mL/min/1.63 m^2^); NA-DKD: non-albuminuric diabetic kidney disease (UACR < 30 mg/g and eGFR < 60 mL/min/1.63 m^2^); AL-DKD: albuminuric and low estimated glomerular filtration rate diabetic kidney disease (UACR ≥ 30 mg/g and eGFR < 60 mL/min/1.63 m^2^); UACR: urinary albumin to creatine ratio; eGFR: estimated glomerular filtration rate; PWV: pulse wave velocity; AugI: augmentation index; qIMT: quality intima-media thickness; RRI: renal resistivity index; RV/BSA: renal volume/body surface index. *P < 0.05 versus group A; ^†^*P* < 0.05 versus group B; ^‡^*P* < 0.05 versus group C

Furthermore, we found that in NA-DKD patients there was a significantly higher prevalence of carotid atherosclerotic plaque in comparison with controls (59% vs. 31%, *P* = 0.009). No differences were observed between patients with NA-DKD and AL-DKD (59% vs. 74%, *P* = 0.16). In the simple logistic regression analysis, the presence of atherosclerotic plaque was associated with age (OR 1.08, 95% CI 1.01–1.16), eGFR (OR 0.98, 95% CI 0.96–0.99), history of CVD (OR 3.46, 95% CI 1.28–9.35).

Finally, we found increased qIMT in the NA-DKD group compared with controls, without statistical significance (0.83 ± 0.12 mm vs. 0.81 ± 0.14 mm, *P* = 0.60) (Table 2). No differences were observed between patients with A-DKD and controls (0.81 ± 0.14 mm vs. 0.81 ± 0.15 mm, P = 0.91).

### Renal profile of the study population according to UACR and eGFR

The RRI was significantly increased in patients with NA-DKD compared with controls (0.76 ± 0.11 vs. 0.71 ± 0.09,* P* = 0.04) and A-DKD patients (0.76 ± 0.11 vs. 0.71 ± 0.08,* P* = 0.02). Moreover, we found no difference between NA-DKD and AL-DKD patients (0.76 ± 0.11 vs. 0.77 ± 0.08, *P* = 0.51) (Table 2). In the simple regression analysis RRI was associated with age (r = 0.41, *P* < 0.0001), history of CVD (r = 0.18, *P* = 0.04), eGFR (r = − 0.37; *P* < 0.0001), PWV (r = 0.36; *P* < 0.0001).

RV/BSA was significantly lower in NA-DKD vs. A-DKD patients (78.57 ± 17.04 mL/m^2^ vs. 91.45 ± 29.94 mL/m^2^, *P* = 0.04). In addition, RV/BSA was higher in A-DKD patients than in controls, without statistical significance (Table 2).

**Fig. 1:**
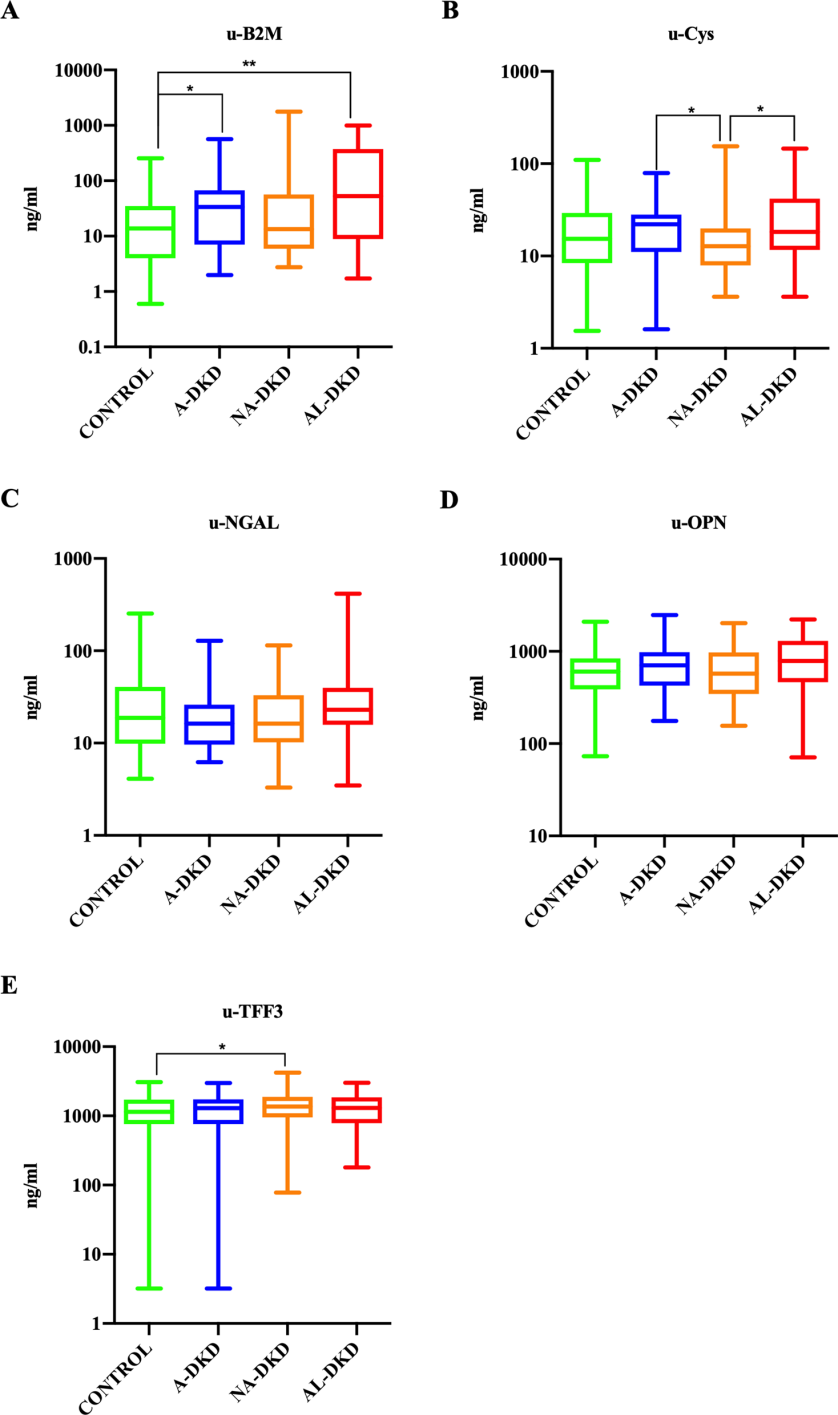
Box plots of urinary biomarkers of kidney injury according to UACR and eGFR. The x-axis shows the study groups, while the y-axis shows the log-transformed concentrations of urinary biomarkers. UACR: urinary albumin to creatine ratio; eGFR: estimated glomerular filtration rate; A-DKD: albuminuric diabetic kidney disease (UACR ≥ 30 mg/g and eGFR ≥ 60 mL/min/1.63 m^2^); NA-DKD: non-albuminuric diabetic kidney disease (UACR < 30 mg/g and eGFR < 60 mL/min/1.63 m^2^); AL-DKD: albuminuric and low estimated glomerular filtration rate diabetic kidney disease (UACR ≥ 30 mg/g and eGFR < 60 mL/min/1.63 m^2^); u-B2M: urinary β2 microglobulin; u-Cys: urinary Cystatin C; u-NGAL: urinary Neutrophil Gelatinase-Associated Lipocalin; u-OPN: urinary osteopontin; u-TFF3: urinary TreFoil Factor 3. *P < 0.05; **P < 0.001

As concerns urinary biomarkers of kidney damage (Fig. 1), u-TFF3 was higher in NA-DKD patients versus controls (1533.14 ± 878.31 ng/mL vs. 1253.84 ± 682.17 ng/mL, *P* = 0.047). In addition, u-Cys was lower in NA-DKD versus A-DKD patients (12.06 [8.09–19.51] ng/mL vs. 22.11 [11.13–27.93] ng/mL, P = 0.01) and AL-DKD patients (12.06 [8.09–19.51] ng/mL vs. 18.20 [11.85–42.93], *P* = 0.01) and u-B2M was higher in AL-DKD patients vs. controls (52.90 [9.06–369.19] ng/mL vs. 13.83 [4.18–33.41] ng/mL, *P* = 0.001) and A-DKD patients versus controls (33.53 [7.71–66.33] ng/mL vs. 13.83 [4.18–33.41] ng/mL, *P* = 0.048). Finally, no statistically significant differences were observed for u-NGAL and u-OPN.

### Multiple regression analysis to identify variables independently associated with PWV, AugI, and RRI variations

We performed a multiple regression analysis (Table 3) in order to identify variables independently associated with PWV and RRI variations.

**Table 3:** Multiple regression analysis evaluating PWV and RRI as dependent variables

	Coefficient β	*P*
PWV		
Multiple regression—Model 1*		
Age, years	0.20	0.0003
Multiple regression—Model 2^†^		
Age, years	0.14	0.02
HbA1c, % (mmol/mol)	0.25	0.008
Multiple regression—Model 3^‡^		
RRI	9.4	0.007
u-OPN, ng/mL	2.6	0.049
RRI		
Multiple regression—Model 1*		
Age, years	0.006	< 0.0001
Multiple regression—Model 2^†^		
Age, years	0.29	0.004
HbA1c, %	0.23	0.007
eGFR, mL/min/1.73 m^2^	− 0.24	0.01
Multiple regression—Model 3^‡^		
Age, years	0.002	0.047
HbA1c, % (mmol/mol)	0.014	0.008
eGFR, mL/min/1.73 m^2^	− 0.01	0.01
u-OPN, ng/mL	0.09	0.006

^*^Model 1 was adjusted for age, sex, BMI, smoking status, SBP, being in secondary prevention. ^†^Model 2 was adjusted for HbA1c, LDL cholesterol, HDL cholesterol, eGFR, UACR > 30 mg/g. ^‡^Model 3 was adjusted for RRI (not when the dependent variable is RRI), u-B2M, u-Cys, u-NGAL, u-OPN, u-TFF3. PWV: pulse wave velocity; AugI: Augmentation Index; BMI: body mass index; SBP: systolic blood pressure; eGFR: estimated Glomerular Filtration Rate; UACR: Urinary Albumin to Creatine Ratio; RRI: renal resistivity index; u-B2M: urinary β2 microglobulin; ; u-Cys: urinary Cystatin C; u-NGAL: urinary Neutrophil Gelatinase-Associated Lipocalin; u-OPN: urinary osteopontin; u-TFF3: urinary TreFoil Factor 3

In the first model PWV showed an association with age (β = 0.20, *P* = 0.0003), while in the second model, the variables that remained significantly related with PWV were age (β = 0.14, *P* = 0.02) and HbA1c (β = 0.25, *P* = 0.008). Finally, in the third model variables that showed a significant association with PWV were RRI (β = 9.4, *P* = 0.02), and u-OPN (β = 2.6, *P* = 0.049).

RRI was associated with age (β = 0.006, *P* < 0.0001) in the first model. Then, in the second model variables that remained significantly related to RRI were age (β = 0.29, *P* = 0.004), HbA1c (β = 0.23, *P* = 0.007), and eGFR (β = − 0.24, *P* = 0.01). Finally, in the third model variables related to RRI were age (β = 0.002, *P* = 0.047), HbA1c (β = 0.014, *P* = 0.008), eGFR (β = − 0.01, *P* = 0.01), and u-OPN (β = 0.09, *P* = 0.006).

### Logistic regression analysis to identify variables independently associated with the presence of carotid atherosclerotic plaque

In the first model, the presence of carotid atherosclerotic plaque was associate with age (OR 1.10, 95% CI 1.01–1.20, *P* = 0.02); while in the second model it was related to history of CVD (OR 4.11, 95% CI 1.08–15.62, *P* = 0.04). Finally, in the third model age remained significantly associated with the presence of carotid atherosclerotic plaque (OR 1.09, 95% CI 1.03–1.18, *P* = 0.04) and the history of CVD (OR 1.93, 95% CI 1.02–6.04, *P* = 0.048).

### Logistic regression analysis to identify variables independently associated with PWV > 10 m/s and RRI > 0.70

In the logistic regression model values of PWV > 10 m/s were associated with age (OR 1.12, 95% CI 1.02–1.23, *P* = 0.01), SBP (OR 1.04 95% CI 1.01–1.07, *P* = 0.02), and HbA1c (OR 2.02, 95% CI 1.31–3.12, *P* = 0.001).

As concerns values of RRI > 0.70, they were significantly associated with SBP (OR 1.04, 95% CI 1.01–1.07, *P* = 0.01), HbA1c (OR 1.61, 95% CI 1.08–2.40, *P* = 0.02), and eGFR (OR 0.97, 95% CI 0.94–0.99, *P* = 0.03].

## Discussion

The increasing clinical relevance of NA-DKD as a distinct phenotype of DKD with its own set of challenges has recently been a topic of growing interest among researchers and clinicians.

In this study, we investigated the cardiovascular risk profile of patients with NA-DKD, using arterial stiffness and qIMT, which are well known early markers of cardiovascular disease and predictive of cardiovascular events. In addition, we investigated the renal injury profile by measuring RRI and dosing urinary biomarkers strictly linked to renal injury.

We found that patients with NA-DKD exhibited higher PWV compared with controls; PWV was similar in NA-DKD patients in comparison with AL-DKD patients. Furthermore, we found a higher prevalence of carotid atherosclerotic plaques in patients with NA-DKD and AL-DKD compared with A-DKD patients and controls.

Previous studies focused on the cardiovascular risk profile of patients with DKD; Kourtidou et al. have recently reported that patients with DKD appear to have higher arterial stiffness and carotid atherosclerosis than patients with type 2 diabetes and preserved kidney function [23]; other authors have reported a relationship between IMT, vascular function, eGFR and stages of diabetic nephropathy [24, 25]. However, none of these studies evaluated markers of cardiovascular disease in different phenotypes of DKD, to identify in which of them the cardiovascular risk is greater. Our results suggest that patients with NA-DKD exhibited a higher risk of developing cardiovascular disease compared with diabetic patients without kidney disease and similar to those with AL-DKD.

These results appear to be in contrast with a recent observational cohort study: Yokohama et al. reported that the risks of death and cardiovascular disease in Japanese patients with type 2 diabetes were not higher in NA-DKD patients than those without renal impairment [26]. Conversely, in line with our data, Penno et al. reported NA-DKD as a strong predictor of mortality [9]. One possible explanation for the inconsistency of these results may be the different rate of macrovascular complications; in fact, the incidence of cardiovascular disease in Japan is significantly lower than in the Caucasian population.

As concerns renal injury profile, we found that patients with NA-DKD exhibited significantly higher RRI compared with controls and A-DKD patients, and similar to those of AL-DKD patients. This is not the first study reporting an increase of RRI in NA-DKD patients: Garofolo et al. found increased RRI in patients with type 2 diabetes and different DKD phenotypes [27]. Accordingly, Afsar et al. have shown higher RRI in patients with both albuminuria and reduced eGFR and a lower RRI in those patients with normoalbuminuria and preserved eGFR [28]. These results are also consistent with previous studies showing a higher RRI in patients with lower eGFR in comparison to those with preserved eGFR [29, 30]. These data demonstrate that increased RRI is present in DKD regardless of albuminuric status; accordingly, it appears to be a clinical feature of NA-DKD. Moreover, we found that RRI ≥ 0.7 inversely correlates with eGFR and directly with SBP and HbA1c in multiple logistic analyses, and we found a relationship between PWV and RRI in multiple regression analyses. These data are consistent with other studies that demonstrated a link between RRI alteration, arterial stiffness and atherosclerosis, with a predictive role for cardiovascular events in diabetic subjects [31, 32].

The increased RRI in NA-DKD is not surprising, considering that RRI may represent a marker of tubulointerstitial injury [33]. In fact, a possible explanation of increased RRI in NA-DKD patients could be the presence of a greater tubulointerstitial rather than glomerular involvement, which is typical of this phenotype [34].

As concerns RV, our results are consistent with another recent study by Garofolo et al. [27], where total renal volume was lower in NA-DKD than in A-DKD patients. Furthermore, the authors reported that the use of 3D US showed no differences than the widely used 2D US in the assessment of RV.

In our study u-TFF3 was higher in NA-DKD patients compared with controls. Interestingly, TFF3 is highly expressed in renal tubules, and it might play a role in epithelial regeneration [35]. Thus, considering the pivotal role of tubular damage in NA-DKD pathogenesis, the increase of this peptide in patients with this specific phenotype of DKD is not surprising. Other studies investigated the role of u-TFF3 in patients with CKD with and without diabetes. Astor et al. showed that u-TFF3 levels were higher in diabetic patients; furthermore, increased u-TFF3 levels were linked to a higher risk of developing DKD [36]. Similarly, Yamanari et al. showed a predictive role of this peptide in CKD progression [37].

No statistically significant differences were observed for u-OPN between different DKD phenotypes. However, in the multiple regression analyses, we found that u-OPN was independently associated with PWV and RRI. OPN is known to be involved in atherosclerosis [38]. In addition, previous studies highlighted a relationship between plasma levels of OPN and arterial stiffness in various clinical scenarios, including patients with coronary artery disease [39] and geriatric subjects [40]. Unfortunately, no studies are available concerning the relationship between u-OPN and arterial stiffness or RRI. Considering that animal studies identified a higher expression of OPN on tubular epithelium and in renal interstitium with a possible role in DKD progression [41], the association of urinary levels of this molecule and arterial stiffness and RRI in this field is justified.

This study presents some strengths and limitations. As concern strengths, we tried to obtain a complete cardiovascular and renal profile of different DKD phenotypes, which is still an underexplored field. In addition, we used well validated measurements, such as PWV, qIMT, the presence of atherosclerotic plaques, and RRI.

On the other hand, this was a cross-sectional study, thus a longitudinal causal relationship cannot be established. In addition, study groups presented different sex distributions.

## Conclusions

In conclusion, our study analyzed the differences existing in various DKD phenotypes, highlighting the possible central role of eGFR in the definition of cardiovascular risk profile of diabetic patients over the more studied albuminuria. The NA-DKD phenotype presents a clinical challenge in early diagnosis and management for its implications not only for kidney-specific therapy, but also for cardiovascular risk. Thus, a timely diagnosis and a multidisciplinary management should be pursued to optimize global patient outcomes.

## Data Availability

The dataset used and/or analysed during the current study is available from the corresponding author on reasonable request.

## Abbreviations

ACEi: Angiotensin coverting enzyme inhibitors
A-DKD: Albuminuric diabetic kidney disease
AL-DKD: Albuminuric and low estimate glomerular filtration rate diabetic kidney disease
BMI: Body mass index
BP: Blood pressure
BSA: Body surface area
CKD: Chronic kidney disease
CKD-EPI: Chronic Kidney Disease Epidemiology Collaboration
CVD: Cardiovascular disease
DKD: Diabetic kidney disease
eGFR: Estimated glomerular filtration rate
NA-DKD: Non-albuminuric diabetic kidney disease
PWV: Pulse wave velocity
qIMT: Quality intima-media thickness
RRI: Renal resistive index
RV: Renal volume
SBP: Systolic blood pressure
SGLT-2i: Sodium-glucose cotransporert-2 inhibitors
UACR: Urinary albumin to creatinine ratio
u-B2M: Urinary β2-microglobulin
u-Cys: Urinary cystatin C
u-NGAL: Urinary neutrophil gelatinase-associated lipocalin
u-OPN: Urinary osteopontin
u-TFF3: Urinary trefoil factor 3
VIF: Variance inflation factor
